# What are the key conditions associated with lower limb amputations in a major Australian teaching hospital?

**DOI:** 10.1186/1757-1146-5-12

**Published:** 2012-05-30

**Authors:** Peter A Lazzarini, Sharon R O’Rourke, Anthony W Russell, Damien Clark, Suzanne S Kuys

**Affiliations:** 1 Allied Health Research Collaborative, Metro North Health Service District & Queensland University of Technology, The Prince Charles Hospital, Rode Road, Chermside QLD, 4032 Brisbane, Australia; 2 Department of Podiatry, Metro North Health Service District, Queensland Health, Brisbane, Australia; 3 School of Clinical Sciences, Queensland University of Technology, Brisbane, Australia; 4 Cairns Diabetes Centre, Cairns and Hinterland Health Service District, Queensland Health, Cairns, Australia; 5 Department of Diabetes & Endocrinology, Princess Alexandra Hospital, Queensland Health, Brisbane, Australia; 6 School of Medicine, University of Queensland, Brisbane, Australia; 7 Musculoskeletal Research Program, Griffith Health Institute, Griffith University, Gold Coast, Australia

**Keywords:** Amputation, Foot, Diabetes, Peripheral arterial disease, Trauma

## Abstract

**Background:**

Lower extremity amputation results in significant global morbidity and mortality. Australia appears to have a paucity of studies investigating lower extremity amputation. The primary aim of this retrospective study was to investigate key conditions associated with lower extremity amputations in an Australian population. Secondary objectives were to determine the influence of age and sex on lower extremity amputations, and the reliability of hospital coded amputations.

**Methods:**

Lower extremity amputation cases performed at the Princess Alexandra Hospital (Brisbane, Australia) between July 2006 and June 2007 were identified through the relevant hospital discharge dataset (n = 197). All eligible clinical records were interrogated for age, sex, key condition associated with amputation, amputation site, first ever amputation status and the accuracy of the original hospital coding. Exclusion criteria included records unavailable for audit and cases where the key condition was unable to be determined. Chi-squared, t-tests, ANOVA and post hoc tests were used to determine differences between groups. Kappa statistics were used to measure reliability between coded and audited amputations. A minimum significance level of *p* < 0.05 was used throughout.

**Results:**

One hundred and eighty-six cases were eligible and audited. Overall 69% were male, 56% were first amputations, 54% were major amputations, and mean age was 62 ± 16 years. Key conditions associated included type 2 diabetes (53%), peripheral arterial disease (non-diabetes) (18%), trauma (8%), type 1 diabetes (7%) and malignant tumours (5%). Differences in ages at amputation were associated with trauma 36 ± 10 years, type 1 diabetes 52 ± 12 years and type 2 diabetes 67 ± 10 years (*p* < 0.01). Reliability of original hospital coding was high with Kappa values over 0.8 for all variables.

**Conclusions:**

This study, the first in over 20 years to report on all levels of lower extremity amputations in Australia, found that people undergoing amputation are more likely to be older, male and have diabetes. It is recommended that large prospective studies are implemented and national lower extremity amputation rates are established to address the large preventable burden of lower extremity amputation in Australia.

## Background

Lower extremity amputation results in significant global morbidity and mortality [1-11]. The global annual incidence of amputation ranges from 3 per 100,000 populations in Spain and Japan to 44 people per 100,000 in American Indian populations [1,10]. However, a recent 2011 review suggests global ranges of 6 to 31 per 100,000 in Italian and German populations respectively [11]. Mortality rates for lower extremity amputations are reported to be higher than some cancers’ mortality rates [12]; in hospital mortality ranges between 2 – 19% [3,4,9,13], one-year mortality between 10 – 52% [4,7,13], and up to 80% mortality at five years [2,7,12,13].

Amputations are usually the result of complications of diabetes, peripheral arterial disease, trauma, and malignant tumours; and are often complicated by infection [1,3,5,10,14]. Diabetes complications are commonly acknowledged as the leading cause of the global amputation burden and contribute to between 25% (in Italy and Japan) and 90% (in American Indians) of all amputations [1]. In the UK and Europe diabetes accounts for around 40 - 64% of amputations [4,9,11,15]. Peripheral arterial disease is a contributing cause for between 16 – 100% of global amputations [1], and a primary cause (without diabetes or non-diabetes) for 18 – 58% of amputations in the UK and European countries [5,11,14,16]. Amputations related to trauma result in between 0 – 57% of all global amputations and trauma appears to be the primary cause of 2 – 13% of UK and European amputations [3,5]. Finally, malignant tumours are a contributing cause of up to 14% of amputations [17,18] and a primary cause of between 2 – 3% of amputations in the UK and Europe [3,5,16]. Infections contribute to anywhere between 4 -100% of all amputations, however infections are typically preceded by the above conditions [1,11,16]

There appears to be very few Australian studies reporting lower extremity amputations that are associated with any condition; and not solely diabetes [13,19]. The last Australian study reporting on all levels of lower extremity amputations, published in 1990, suggested annual amputation rates ranged between 20 to 28 per 100,000 from 1981 – 1985 in different Australian states [19]. The major causes of amputation identified at that time were vascular disease, trauma, joint disorders, malignancies and infection [19]; however the relative impact of those conditions on amputations was not reported. Interestingly this study did not mention diabetes as a cause of amputation. A more recent Australian study of only major lower extremity amputations in a large tertiary hospital vascular surgery department found 50% of amputations were associated with diabetes [13] supporting the national guideline statement that “half of all non-traumatic amputations are performed on people with diabetes” [20]. Other causes contributing to major amputations included peripheral arterial disease (76%), infection (20%), and trauma (3%) [13]. Interestingly patients undergoing amputation had considerable risk factors for chronic disease such as hypertension (77%), hypercholesterolemia (29%), raised serum creatinine (35%) and/or a history of smoking (82%) [13].

Reports on diabetes only lower extremity amputation in Australia are slightly more prevalent and appear to demonstrate high national rates in comparison to other industrialised nations [21-24]. For example, reports indicate a median annual diabetes-related lower extremity amputation rate for industrialised nations of 12 per 100,000 populations [21]. However, Australian national diabetes-related amputation data, captured in 2004/05, suggested an Australian amputation rate of 17 per 100,000 [22] and a recent New South Wales report indicated a similar diabetes-amputation rate of 18 per 100,000 in 2007 [23]. Regardless, it would appear that Australian diabetes amputation rates are increasing, up from the 14 per 100,000 previously reported in 1997/98 [24]. A Victorian study, suggests these figures may still be a significant underestimate identifying that only one third of the actual diabetes-related foot complications, that often result in amputation, were captured correctly using standard Australian hospital coding [25]. These, and other reports, indicate that diabetes-related amputation in Australia continues to consume significant hospital resources including average length of stays between 25 and 26 days [22,24] and direct costs of $26,700 per amputation [26].

Lower extremity amputation rates appear to becoming more important in analysing health care as they are increasingly used as a marker of the quality and overall structure of health care services; particularly in diabetes [3,4,21,23,27]. A low amputation rate may indicate lower diabetes prevalence, interested health professionals, or accessible, quality integrated health care services; while conversely a high amputation rate may be indicative of higher diabetes prevalence, social disadvantage, particularly interventionist health professionals or inaccessible and uncoordinated health care services [27]. Studies consistently demonstrate that best practice foot complication management utilising multidisciplinary foot teams and well structured and integrated health care services can significantly reduce amputation rates; particularly in diabetes and peripheral arterial disease populations [9-11,14,15,28-31].

For such a seemingly large and preventable cause of morbidity and mortality, the reporting of lower extremity amputation causes and rates in Australia appears to be a largely under-developed area of research. To the best of the authors’ knowledge, this study is expected to be the first in over twenty years to report on key conditions associated with all levels of lower extremity amputation in an Australian population. Furthermore, the authors aimed to take the opportunity to examine the reliability of existing Australian hospital discharge datasets reporting of lower extremity amputations to add to the existing Australian literature in the area [25,32,33]. The findings of this study may assist clinicians’ and policy-makers’ understandings of the major conditions causing lower extremity amputations in Australia, the accuracy of existing data collection systems for lower extremity procedures, and how best to direct services to address the large burden of amputations in Australia.

The primary aim of this retrospective study was to investigate the key conditions associated with lower extremity amputations in a major Australian tertiary teaching and referral hospital. Secondary objectives were to determine the influence of age and sex on lower extremity amputations, and the reliability of lower extremity amputation coding in hospital discharge datasets.

## Methods

### Setting and participants

The study was a retrospective analysis of the clinical records of patients who had undergone a lower extremity amputation procedure at the Princess Alexandra Hospital, Brisbane Australia between 1^st^ July 2006 and 30^th^ June 2007. The Princess Alexandra Hospital was chosen as it is the major tertiary teaching and referral hospital for southern Queensland, Australia; servicing 1.5 million people across the Metro South, Darling Downs–West Moreton and South West Queensland Health Service Districts. It also houses the only vascular surgery department and Queensland Amputee Limb services for the same southern region which makes up one third of the population of Queensland, Australia. Queensland Health data indicated that in 2006/07 the Princess Alexandra Hospital performed 21% of all lower extremity amputations performed in Queensland [34]. The Human Research Ethics Committee at the Princess Alexandra Hospital, Metro South Health Service District, Brisbane, Australia provided ethical approval for the study.

### Procedure

Eligible participants were identified via a request to the Princess Alexandra Hospital Health Information Management Service to obtain a report on all lower extremity amputation procedures performed at the Princess Alexandra Hospital between 1^st^ July 2006 and 30^th^ June 2007. The report was generated by identifying, including and listing all patients that had International Classification of Diseases (ICD-10-AM) codes that described a lower extremity amputation procedure [35]. Furthermore, it was requested that the report specify if each identified amputation case also had an associated ICD code for a diagnosis of diabetes or trauma to further determine the reliability of coding for these variables or coded diagnoses [35]. The conditions of diabetes and trauma were chosen as highly representative of key conditions that are associated with lower extremity amputations in the literature [1,3,5,10,11,14]. Table 1 displays the ICD-10-AM (5th Edition) codes used to identify and retrieve the information for this report.

**Table 1:** International Classification of Diseases (ICD-10-AM) codes identified for lower extremity amputation, diabetes and trauma

**Category**	**Sub-category**	**ICD codes**	**ICD Sub-codes**	**ICD description**
Amputation	Minor	1533		Amputation of ankle or foot
			44338-00	Amputation of toe
			44358-00	Amputation of toe including metatarsal bone
			90557-00	Disarticulation through toe
			44361-00	Disarticulation through ankle
			44364-00	Midtarsal amputation
			44364-01	Transmetatarsal amputation
			44361-01	Amputation of ankle through malleoli of tibia and fibula
	Major	1505		Other excision procedures on knee or leg
			44637-01	Disarticulation at knee
			44367-02	Amputation below knee
	Major	1484		Amputation of pelvis or hip
			44370-00	Amputation at hip
			44373-00	Hindquarter amputation
			44367-00	Amputation above knee
Diabetes		E09 – E14		Presence of a diabetes code including Impaired Glucose Regulation
Trauma		S00 – T79.99		Episodes with trauma as the reason for admission

All eligible participants’ charts were systematically interrogated for the following variables: age, sex, amputation site, first amputation status, diabetes status, trauma status, and any other key condition associated with the amputation. The audit procedure initially involved capturing age (years), sex, first ever amputation status (yes or no) and amputation site. Amputation site was defined as either amputation of a digit/s, ray/s (included Metatarsal/s), mid-foot, below knee or above knee. These sites were also grouped into minor (digit/s, ray/s or mid-foot) or major (below knee or above knee) amputations as defined in accepted international clinical guidelines [2], ICD-10-AM codes [35] and many other similar amputation studies [3-5,11,13,36].

The chart was then further interrogated for a diagnosis of diabetes (type 1, type 2, or non-diabetes) and trauma of the foot or lower extremity (yes or no). If a diagnosis of diabetes or trauma was established this was recorded as the key condition associated with the amputation and this section of the audit would cease. If neither diabetes nor trauma was evident further interrogation of the chart was undertaken to determine the most likely cause of amputation according to key conditions reported to precipitate amputation; including peripheral arterial disease (non-diabetes), peripheral neuropathy (non-diabetes), orthopaedic lower limb joint deformity (non-diabetes) or malignant tumours (non-diabetes) [1,3-5,10,11,14,20]. As foot ulcers and infection precipitate most amputations and diabetes, peripheral arterial disease and peripheral neuropathy are the predominant causes of foot ulcers and any resultant infection [1,10,14,20]; it was decided not to capture these as key conditions or risk factors associated with amputation unless no other key condition was evident.

Lastly, audited data on diabetes status, trauma status and amputation site on each case was compared to the original standard hospital discharge dataset coded case to determine the reliability of the original hospital coded data for these variables. Accuracy was recorded when the original coded case was in agreement with the audited results for each case, and overall, for variables of diabetes status, trauma status and amputation site. Exclusion criteria for the study included any cases whose clinical records were unavailable during the audit procedure period or whose singular key condition for amputation was unable to be determined.

### Statistical analysis

Data was analysed using SPSS 18.0 for Windows (SPSS Inc., Chicago, IL, USA). Descriptive statistics were used to display single variable quantities using means and standard deviations (SD) for continuous variables or proportions for categorical variables unless otherwise indicated. Chi-squared tests were used to test differences in sex, first amputation status and amputation site groups within total amputations or key condition groups. Independent t-tests were used for testing differences in mean age at amputation for sex, first amputation and amputation site groups. One-way analysis of variance (ANOVA) and post hoc tests were used for testing differences in mean age at amputation for different key condition groups. The proportion of total amputations of each key condition group was calculated with 95% confidence intervals. Kappa (*K)* statistics and percentage agreement were used to test the measurement of agreement between the original hospital coded results and the audited results. The *K* statistic or value has a maximum score of 1 for overall perfect agreement and a minimum score of 0 or less for poor agreement [37]. The 95% confidence intervals for the *K* value were generated using GraphPad Software. A minimum significance level of *p* < 0.05 was used throughout.

## Results

One hundred and ninety seven eligible cases of lower extremity amputation were identified for the 2006/07 period. Eleven cases were excluded due to the unavailability of clinical records during the audit procedure period (n = 9) or whose singular key condition for amputation was unable to be determined (n = 2). Thus, 186 cases met the inclusion criteria and were audited.

Patients ranged in age from 18 to 100 years old (62 ± 16 (mean ± SD)). Male amputations accounted for 129 (69%) amputations which was significantly higher than females (31%) (*p* < 0.001). Of the 186 included cases: 160 (86%) were unique patients, 104 (56%) recorded their first ever amputation and 101 (54%) underwent major amputations (or a major : minor amputation ratio of 1.19:1). The breakdown of overall specific amputation sites revealed 57 (31%) above knee, 56 (30%) digit, 44 (24%) below knee, 19 (10%) ray and 10 (5%) mid-foot or trans-metatarsal amputations.

**Figure 1:**
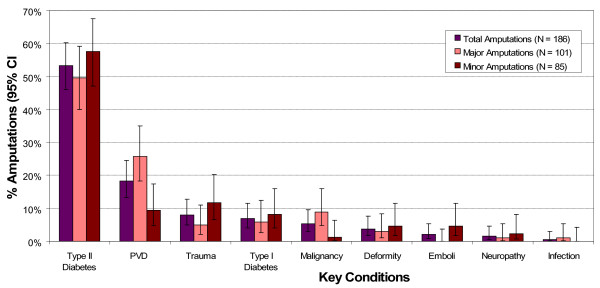
Proportion of key conditions associated with lower extremity amputations (minor, major and total amputations).

Key conditions associated with lower extremity amputation identified by this study included type 1 diabetes, type 2 diabetes, trauma (non-diabetes), peripheral arterial disease (non-diabetes), malignant tumours (non-diabetes), orthopaedic joint deformity (non-diabetes), post-surgical emboli (“trash toes”) (non-diabetes), neuropathy (non-diabetes), and infection (non-diabetes). Table 2 displays the descriptive statistics of numbers, proportions (%), mean age (SD), and minimum and maximum ages for each key condition associated with lower extremity amputations. There were significant differences between the proportion of type 2 diabetes (53.2%) and all other key conditions and peripheral arterial disease (non-diabetes) (18.3%) and all other key conditions (*p* < 0.05). Figure 1 displays the proportions of individual key conditions associated with amputation for all minor amputations, major amputations and total amputations (with 95% confidence intervals).

**Table 2:** Numbers, proportions (%), mean age (SD), minimum and maximum ages of key conditions associated with lower extremity amputation

	**Number**	**%**	**Mean age**	**SD**	**Min**	**Max**
Type 2 Diabetes	99	53.2	67	10	41	94
PAD	34	18.3	68	18	26	100
Trauma	15	8.1	36	10	18	54
Type 1 Diabetes	13	7.0	52	12	34	67
Malignant tumours	10	5.4	56	19	23	85
Joint Deformity	7	3.8	55	19	31	89
Emboli	4	2.1	69	6	62	74
Neuropathy	3	1.6	48	8	40	55
Infection	1	0.5	84	84	-	-
Total	186	100	62	16	18	100

PAD = Peripheral arterial disease (non-diabetes).

Table 2 also displayed differences in the mean age at amputation for groups of key conditions with at least 10 recorded cases (F = 23.057, *p* < 0.001). Post hoc tests revealed this difference in age remained significant between the trauma group 36 ± 10 yrs and all other groups (*p* < 0.01); between type 1 diabetes 52 ± 12 yrs and type 2 diabetes 67 ±10 yrs (*p* < 0.01); and between type 1 diabetes and peripheral arterial disease (non-diabetes) 68 ± 18 yrs (*p* < 0.01). The overall mean age at amputation was different for males (60 ± 16 yrs) and females (67 ± 14 yrs) (*p* < 0.01). There were no statistically significant age differences overall between first amputation status (62 ± 18 yrs) and subsequent amputation status (63 ± 13 yrs) or major amputation (63 ± 17 yrs) and minor amputation (61 ± 15 yrs).

Table 3 displays the numbers, proportions (%) and *p* Values for differences in sex, first amputation and major amputation status for each key condition associated with lower extremity amputation. The trauma group demonstrated that all amputations primarily associated with trauma (n = 15) were first ever amputations and only occurred in males (*p* < 0.01). In the malignant tumours group (n = 10) there were more females (60%) undergoing amputation and more major amputations (90%) compared to minor amputations (10%) (*p* < 0.05). Also there were significantly more major amputations (76.5%) than minor (23.5%) amputations in the peripheral arterial disease (non-diabetes) group (n = 34) (*p* < 0.01).

**Table 3:** **Numbers (n), proportions (%) and *p *Values for sex, first amputation and amputation site groups within key conditions associated with lower extremity amputation**

	**Male sex**		**First amputation**		**Major amputation**	
	**n (%)**	** *p * value**	**n (%)**	** *p * value**	**n (%)**	** *p * value**
Type 2 Diabetes	65 (65.7)	0.243 (NS)	51 (51.5)	0.197 (NS)	50 (50.5)	0.268 (NS)
PAD	25 (73.5)	0.559 (NS)	17 (50.0)	0.442 (NS)	26 (76.5)	0.004
Trauma	15 (100)	0.007	15 (100)	< 0.001	5 (33.3)	0.089 (NS)
Type 1 Diabetes	9 (69.2)	0.997 (NS)	4 (30.8)	0.061 (NS)	6 (46.2)	0.526 (NS)
Malignant tumours	4 (40.0)	0.038	6 (60.0)	0.789 (NS)	9 (90.0)	0.020
Joint Deformity	5 (71.4)	NA	6 (85.7)	NA	3 (42.9)	NA
Emboli	3 (75.0)	NA	2 (50.0)	NA	0 (0)	NA
Neuropathy	3 (100)	NA	2 (66.7)	NA	1 (33.3)	NA
Infection	0 (0)	NA	1 (100)	NA	1 (0)	NA
Total	129 (69.4)	< 0.001	104 (55.9)	0.107 (NS)	101 (54.3)	0.241(NS)

PAD = Peripheral arterial disease (non-diabetes).

NS = Not significant.

NA = Not applicable to test as the assumption of Chi-Squared test is violated as 2 cells had expected counts of < 5.

Table 4 displays the reliability or measurement of agreement of hospital coded amputations compared with the audited records. Agreement for the individual variables of diabetes status, trauma status and amputation site were rated as almost perfect (*K* > 0.81) according to the Kappa strength of agreement categories [37]. The overall proportion of original hospital coded amputations that were completely accurate according to the audit records for all three variables was 93.0%.

**Table 4:** Measure of agreement between hospital coded amputation status and audited amputation status

	**Agreement (%)**	** *K * value (95% CI)**	**Strength of agreement **[37]
Diabetes Status	97.8	0.95 (0.91 – 1.00)	Almost perfect
Trauma Status	97.3	0.81 (0.65 – 0.97)	Almost perfect
Amputation Site Status	98.4	0.98 (0.95 – 1.00)	Almost perfect
Overall	93.0	N/A	N/A

## Discussion

This study appears to be the first in over 20 years to investigate a large series of patients undergoing any level of lower extremity amputation in an Australian population [19]. The findings align with international studies indicating that people undergoing amputations were more likely to be older (mean 62 years), male (by a ratio of 2:1) and have diabetes or peripheral arterial disease (approximately 80%) [1,4,5,9,13,14,16]. Also the major to minor amputation ratio (1.19:1) seems to be consistent with international literature [1,10,36].

Whilst, this study’s results align mostly with those already reported in the international literature, it did identify some interesting findings with regard to: proportions of key conditions associated with amputation, mean age at amputation for key conditions, differences within key conditions, and first amputation status.

The proportions of key conditions associated with amputations in this study were mostly within ranges reported in existing international literature [1,4,5,9,14]. Although, interestingly the major key condition associated with lower extremity amputation found in this study, diabetes, was not reported as a key condition associated with lower extremity amputation in the last similar Australian study published in 1990 [19]. The proportions of diabetes-related (type 1 and type 2) (60.2%) and malignancy-related (5.4%) amputations appear to be disproportionately high compared to the prevalence of these conditions in the general Australian population (diabetes (4.0%) and malignancy (1.6%)) [38]. Furthermore, diabetes-related amputations seem to remain disproportionately high even when compared with similar ‘higher risk’ populations observed in this study; i.e. in adults (> 18 years) with multiple comorbidities in Australia (diabetes prevalence 5.3 – 19.2%) [39], yet, malignancy-related amputations become more proportionate in those ‘higher risk’ populations (malignancy prevalence 3.2 – 7.0%) [39]. In contrast, the proportions of peripheral arterial disease-related (18.3%) and trauma-related (8.1%) amputations seem to be proportionate to the prevalence of similar conditions in the general Australia population (cardiovascular disease (16%) and injuries (12.0%) [38] and even in the ‘higher risk’ populations (cardiovascular disease 6.1 – 20.9) [39].

Furthermore, this study identified that around 80% of amputation cases were associated with complications of conditions defined by the Australian Institute of Health and Welfare, and World Health Organisation to be preventable chronic diseases (diabetes, peripheral arterial disease and malignancy) [40,41]. Additionally, nearly 10% resulted from trauma or accidents. Thus, it may be suggested that most amputations are associated with a potentially preventable condition, complication or circumstance.

Age at amputation was significantly different for different groups of key conditions. Amputations associated with type 1 diabetes in our study occurred at significantly younger ages (52 ± 12 years) than type 2 diabetes (67 ± 10 years) or peripheral arterial disease (68 ± 18 yrs). Younger age has previously been reported for amputations due to type 1 diabetes (45 – 62 years) [4,9,31], compared to amputations due to type 2 diabetes (68 – 73 years) [4,9,14], and peripheral arterial disease (70 – 79 years) [9,14]. Furthermore, the proportion of amputations due to type 1 diabetes made up approximately 10% of the total diabetes associated amputations in this study, which is comparable to previous literature [4,9]. This may be associated with the different age of onset for the diagnoses of these various conditions [38] and a similar proportion of the total population of people with diabetes having type 1 compared with type 2 diabetes [22]. Our findings suggest that having type 2 diabetes does not confer any additional risk for amputation compared with type 1 diabetes. Not surprisingly, amputations associated with trauma demonstrated a much younger age of onset (36 ± 10 yrs) than the average age of onset for all lower extremity amputation (62 ± 16).

Differences within key conditions associated with lower extremity amputation included that patients having an amputation due to malignant tumours were more likely to be female and need a major amputation [5]. However, the numbers included in this group were small, and included six carcinomas and four sarcomas, thus, the small numbers and differing development of these different categories of malignant tumours should be interpreted with caution. All amputations due to trauma were first amputations, only occurred in male participants and were frequently caused by motor vehicle and work place accidents, which aligned with similar findings from the literature [5]. Lastly, significantly more major amputations occurred in the peripheral arterial disease (non-diabetes) group which reflects existing international literature [1] and the more proximal nature of the disease that occurs in patients suffering from non-diabetes peripheral arterial disease [6].

The results for the proportion of first ever amputation (56%) in this overall series, however, seems to be at the lower range of those previously reported (50 – 86%) [1,10,13]. This may be the result of investigating a major tertiary referral hospital that houses the only vascular surgery department for a geographically vast region containing over 35 other hospitals in southern Queensland. Anecdotally, the authors observed in the clinical records many historical first amputations occurring in the patients’ local hospital with more complex cases requiring amputation or subsequent amputation procedures being transferred to the Princess Alexandra Hospital for more specialist care.

Lastly, this study found that hospital coding reliability for variables associated with lower extremity amputation (diabetes status, trauma status and amputation site) were categorised as almost perfect; above 0.8 Kappa values or 90% across all variables tested. This result seems to align with other methodologically similar Australian studies and adds further weight to the reliability of Australian discharge datasets in collecting data around lower extremity amputation procedures [32,33]. However, these results are in contrast to a previous Australian study investigating the hospital coding accuracy of diabetic foot complication pathology which reported an accuracy rate as low as 34% [25]. This result is not entirely unexpected as the previous study had significant methodological differences and its primary focus was diabetic foot pathology and not specifically amputations as was the primary focus of this study [25].

### Limitations

There are a number of methodological differences in this study when compared to other similar studies. Thus, the comparative results of this study should be viewed with some caution. Firstly, this study did not follow the protocol for such audits as defined by the Global Lower Extremity Study Group [1]. This group recommend that at least two data sources are used to cross-check accuracy of data and estimate levels of case ascertainment; including hospital discharge data, operating theatre records, limb fitting centre records, amputation registries, and/or foot clinic records [1]. Our study only used the one source from coded hospital discharge dataset records as other sources were not available or accessible at the time. This limitation may also impact our findings on the reliability of hospital coding as only those amputations originally coded have been audited and additional amputations that may have been missed by hospital coders remain unknown as identified in other studies [25]. However, further Australian study’s’ findings investigating the reliability of lower extremity amputation coding reflected our results, potentially due to the more formal standard documentation that occurs for a surgical procedure than clinical assessment [32,33].

Secondly, the definition of major amputations used in this study was different to that defined by the Global Study Group. The group defines a major amputation as that through, or proximal to, the tarso-metatarsal joint [1]. However, the definitions used in our study aligned with those in commonly used international clinical guidelines [2], ICD-10-AM codes [35] and other similar large studies [3-5,11,13,36] defining a major amputation being a resection proximal too, not through, the mid-tarsal level. This may have contributed to our reported lower proportions of major amputations compared to studies that have used different definitions of major and minor amputations [1,10].

Thirdly, our study only interrogated medical records for a single principle key condition precipitating the amputation, thus, multiple key conditions were not recorded for each case as performed by other studies [1,10,13]. Also this study did not use standard co-morbidity measures to capture and analyse co-morbidities [42] and instead reported those conditions already stated in the literature to increase the risk of amputation rather than mortality as per other similar international studies [1,3,5,10,20]. Thus, other standard co-morbidities were not captured like other Australian studies have done; for example, those that are risk factors for chronic diseases like hypertension, dyslipidaemia or long term smoking [13]. These conditions were frequently observed throughout this case series.

Fourthly, our study prioritised the diagnosis of diabetes over other conditions associated with amputations and this may have potentially over inflated diabetes as the key condition association with amputation. However, other studies using similar methodology to ours in this regard found similar results [3,5]. Lastly, typical of a single site, retrospective study the quality of data obtained from medical records can lack rigour and generalisability. However, the site used in this study may provide more generalisable results than otherwise, as it was the major tertiary referral site for a third of the population of Queensland, performs over 20% of all Queensland lower extremity amputations [34], and its resultant amputation rate of 13.1 per 100,000 for the 1.5 million people it services [34] is comparable to that of international amputation rates [1,11].

Whilst these differences may decrease comparability with some other studies, the results of this study align with the results obtained in most international studies. At the very least, robust data on the proportions of key conditions associated with all levels of lower extremity amputations in Australia, mean age at amputation, sex influence, and amputation site ratios is provided by this study. However, it would be recommended that for any future Australian studies investigating lower extremity amputations that the Global Study Group protocol be employed prospectively across multiple sites to improve the rigour and generalisability of results; with the exception of aligning amputation definitions with standard ICD code definitions and international guidelines.

Finally, this study supports an existing Australian recommendation stating that the reporting of standard annual national lower extremity amputation rates should be implemented in Australia [43]; including categories associated with at least type 1, type 2 and non-diabetes amputations [4,9]. The reporting of these rates are best practice in other industrialised countries and have contributed to the reduction of both diabetes and non-diabetes amputations in these nations [1,3,4,10,11,14,15,21,28,29]. The authors suggest that until such a standard lower extremity rate is reported across Australia the old adage of “you can’t improve what you can’t measure” will continue for this highly preventable complication.

## Conclusions

This study appears to be the first in over 20 years to report on the key conditions associated with all levels of lower extremity amputations in an Australian population. The results are consistent with those displayed in the international literature for age, sex, amputation sites and the strong association with chronic diseases. Differences in age between amputations associated with trauma, type 1 and type 2 diabetes were noted. The strong association with chronic diseases emphasises the need for routine screening and management of the high risk foot in order to reduce amputation rates; in particular in patients with diabetes. It is also recommended that large, standardised, prospective multi-site studies and standard national reporting of lower extremity amputation rates are established in Australia. Only with these standard rates implemented, reported and investigated will Australia truly be able to address the large preventable burden of morbidity and mortality caused by lower extremity amputation.

## Competing interests

The authors have no relevant conflict of interest to disclose.

## Authors’ contributions

PAL conceived, designed, researched data, contributed to discussion, wrote and reviewed/edited the manuscript. SRO conceived, contributed to discussion, and reviewed/edited the manuscript. AWR and DC contributed to discussion and reviewed/edited the manuscript. SSK researched data, contributed to discussion, wrote and reviewed/edited the manuscript. All authors read and approved the final manuscript.
